# Successful Extirpation of Cancer of the Rectum

**Published:** 1831

**Authors:** 


### SUCCESSFUL EXTIRPATION OF CANCER OF THE RECTUM.

THIS operation took place at the Versailes hospital, and was performed by M. Maurin. The patient (J. Baptiste) was 31 years of age, of delicate constitution, who presented himself to M. Maurin in the beginning of September, 1828, complaining that he could not procure a stool, except by the aid of enemata; and that he felt great weight and acute pains in the rectum. On examination by the finger, a hard and irregular tumour was discovered, about two inches from the anus, ulcerated in the centre, and discharging a sanious ichor, of a most intolerable fetor. The mobility of the tumour, notwithstanding its distance from the orifice of the gut, induced the surgeon to entertain the idea of extirpation. Baron Dupuytren was consulted on the 17th September, and made the following note. "There exists about two inches from the orifice of the rectum a carcinomatous tumour, occupying one side of the gut to the extent of about two inches. There is no chance of a cure, except by an operation—and this operation must be both difficult and dangerous. If the patient shall make up his mind to the risk of the operation, I am ready to attempt it." *Sept.* 21*st.* Encouraged by this opinion of so celebrated a surgeon, M. Maurin himself determined to operate. For this purpose he made an incision through the posterior and left part of the sphincter, by means of a probe-pointed bistoury, when the tumour was seized by a kind of tenaculum, and drawn downwards gradually and gently, till it appeared in view, when it was carefully removed by means of scissors. When taken out, it was found to be of an oval form, a little flattened, and two inches in length, with an ulceration on one side. It was of a very compact tissue. The operation was very painful, and considerable hæmorrhage attended, but was soon arrested by stuffing the rectum. In the course of five hours after the operation the patient experienced acute pains in the epigastrium, with dysury, sharp fever, and intense thirst. Two bleedings relieved these symptoms, and he slept some in the course of the night. In the morning the pulse was reduced from 140 to 100, and the patient was again bled. When the dressings were removed, there issued a considerable quantity of pus with blood. In the course of the succeeding days the state of the patient was improved—the purulent discharge lessened—the lancinating pains ceased, and, by the 15th November, the discharge was almost nothing. Consistent and spontaneous stools were passed. On the 1st December the wound in the sphincter was found to be cicatrized. On the 8th of the same month, the patient was discharged from the hospital cured. He experienced no difficulty or pain in passing his motions.

This case, taken in connexion with that reported in the present PORTFOLIO, from the Middlesex Hospital, holds out some hope in circumstances where the patient's life is rendered miserable by a painful disease, that seems to admit of no other remedy but the knife.—REVUE MED. *Feb.* 1831.

---

